# Predicting the probability of Gaucher disease in subjects with splenomegaly and thrombocytopenia

**DOI:** 10.1038/s41598-021-82296-z

**Published:** 2021-01-28

**Authors:** Irene Motta, Dario Consonni, Marina Stroppiano, Christian Benedetto, Elena Cassinerio, Barbara Tappino, Paola Ranalli, Lorenza Borin, Luca Facchini, Andrea Patriarca, Wilma Barcellini, Federica Lanza, Mirella Filocamo, Maria Domenica Cappellini, Splenomegaly Gaucher group, Francesca Farina, Katia Codeluppi, Elena Rivolti, Federico Simonetti, Francesca Lunghi, Tommasina Perrone, Nicola Sgherza, Valentina Carrai, Anna Maria Cafro, Roberto Cairoli, Angela Amendola, Elena Trabacchi, Daniele Vallisa, Ilaria Burgo, Augusto Bramante Federici, Cecilia Carbone, Mariella D’Adda, Donato Mannina, Valeria Di Giacomo, Giulia Lupparelli, Alessandra Lombardo

**Affiliations:** 1 General Medicine Unit, Rare Diseases Center, Fondazione IRCCS Ca’ Granda Ospedale Maggiore Policlinico, Via F. Sforza, 35, 20122 Milan, Italy; 2 Department of Clinical Sciences and Community Health, Università Degli Studi Di Milano, Milan, Italy; 3 Epidemiology Unit, Fondazione IRCCS Ca’ Granda Ospedale Maggiore Policlinico, Milan, Italy; 4 Laboratorio Di Genetica Molecolare E Biobanche, Istituto G. Gaslini, Genoa, Italy; 5 Università Degli Studi Di Milano, Milan, Italy; 6 Hemophilia and Rare Blood Diseases Centre, Oncology and Hematology Department, S. Spirito Hospital, Pescara, Italy; 7 Hematology Division, San Gerardo Hospital, Monza, Italy; 8 Division of Hematology, Azienda USL-IRCCS Di Reggio Emilia, Reggio Emilia, Italy; 9 Division of Hematology, Department of Translational Medicine, University of Eastern Piedmont and Ospedale Maggiore Della Carità, Novara, Italy; 10 Hematology Unit, Fondazione IRCCS Ca’ Granda Ospedale Maggiore Policlinico, Milan, Italy; 11 UOC Dipartimentale Ematologia, Azienda USL Nord-Ovest Toscana, Ospedale Versilia, Camaiore, Italy; 12 Hematology and Bone Marrow Transplantation Unit, San Raffaele Scientific Institute, IRCCS Milano, Milan, Italy; 13 University of Bari, Bari, Italy; 14 SODc Ematologia Azienda Ospedaliero Universitaria Careggi, Firenze, Italy; 15 Department of Hematology, ASST Grande Ospedale Metropolitano, Niguarda, Milan, Italy; 16 San Carlo Hospital, Potenza, Italy; 17 Hematology, Piacenza, Italy; 18 Hematology and Transfusion Medicine, L Sacco University Hospital, Milan, Italy; 19 Hematology Department, ASST Spedali Civili, Brescia, Italy; 20 Division of Hematology, Azienda Ospedaliera Papardo, Messina, Italy; 21 Hematology Unit, Azienda Ospedaliera Santa Maria di Terni, University of Perugia, Terni, Italy

**Keywords:** Diseases, Haematological diseases

## Abstract

Hematologists are frequently involved in the diagnostic pathway of Gaucher disease type 1 (GD1) patients since they present several hematological signs. However, GD1 is mainly underdiagnosed because of a lack of awareness. In this multicenter study, we combine the use of a diagnostic algorithm with a simple test (β-glucosidase activity on Dried Blood Spot) in order to facilitate the diagnosis in a population presenting to the hematologist with splenomegaly and/or thrombocytopenia associated with other hematological signs. In this high-risk population, the prevalence of GD1 is 3.3%. We propose an equation that predicts the probability of having GD1 according to three parameters that are routinely evaluated: platelet count, ferritin, and transferrin saturation.

## Introduction

Gaucher disease type 1 (GD1) is an autosomal recessive lysosomal storage disorder caused by mutations in the *GBA* gene resulting in the deficiency of β-glucosidase enzyme. Its prevalence in the non-Ashkenazy Jewish population is estimated at 1:40,000–100,000 subjects, whereas in Ashkenazi Jewish is 1:500–1000.

At diagnosis, patients present with several hematological signs and symptoms, including splenomegaly (86%), anemia (64%), thrombocytopenia (56%), bleeding history, and monoclonal gammopathy of undetermined significance (MGUS), leading them to consult a hematologist on their diagnostic pathway^1^. However, an international survey showed that only 20% of hematologists include GD1 in the differential diagnosis of a patient with anemia, thrombocytopenia, hepatomegaly, splenomegaly, and bone pain^2^. As a matter of fact, GD1 is misdiagnosed and underdiagnosed; thus, patients often experience long diagnostic delays, leading to inappropriate procedures, treatments, and complications that often cannot be reversed by the available treatments^2^.

Moreover, half of the patients are diagnosed through bone marrow biopsy, although the diagnostic gold standard is the activity of β-glucosidase on leucocytes or fibroblasts^3^. Among the crucial obstacles to diagnosis, physicians mainly identify outsourced testing and, more importantly, the lack of awareness^4^. Thus, ten years ago, a panel of experts published two diagnostic algorithms, one for the Ashkenazi and one for the non-Ashkenazi Jewish population, to facilitate the diagnosis of GD1 for hematologists^5^.

The new-born screening has been experimented in some areas, showing an incidence of 1:22,205 in Northern Italy^6^. However, the large-scale implementation of new-born screening for a disease with high phenotypic heterogeneity, ranging from asymptomatic to severely symptomatic conditions, should be carefully evaluated.

We hypothesized that an approach that combines a diagnostic algorithm and a simple, cheap, and easy-to-do test could facilitate the diagnosis. We designed a multicenter study that aimed at evaluating the prevalence of GD1 in a high-risk population presenting to the hematologist with splenomegaly and/or thrombocytopenia associated with other hematological signs or symptoms suggestive of GD1. Preliminary results of this study on the first 196 patients have been previously published, showing a GD1 prevalence of 3.6% in a high-risk population^7^.

## Materials and methods

### Study design

We designed a multicenter study among 35 hematology centers in Italy. According to the feasibility questionnaire, we expected to enroll 500 subjects. The enrolment started in September 2010 and closed in December 2018.

Inclusion and exclusion criteria were based on the published algorithm for the non-Ashkenazi population^5^:

- Inclusion criteria: splenomegaly and/or thrombocytopenia and at least one sign or symptom among bone pain history, anemia, MGUS, polyclonal gammopathy in subjects under 30 years of age, splenectomy;
- Exclusion criteria: onco-hematological diseases, portal hypertension due to liver diseases, hemoglobinopathies, or chronic hemolytic anemias.

Demographic, clinical, and laboratory data were collected at enrolment, gathered in a specific case report form, and collected by the coordinating center at Fondazione IRCCS Ca’ Granda Ospedale Maggiore Policlinico, Milan, Italy.

### Beta-glucosidase activity

The beta-glucosidase activity on Dried Blood Spot (DBS) was centralized at Ospedale Gaslini, Genoa, Italy^8,9^. Normal values range from 4.4 to 17.7 pmol/punch/h. Subjects showing activity below 4.4 pmol/punch/h were recalled to be assessed with the gold standard assay on nucleated cell homogenates (leucocytes, EBV-lymphoblasts, or fibroblasts). If the enzymatic defect was confirmed, the diagnosis was completed with the molecular *GBA* analysis.

### Statistical analysis

The prevalence of GD1 and its 95% confidence interval (CI) based on the exact method were calculated. Demographic, clinical, and laboratory variables of patients affected by GD1 were compared to those of unaffected patients using Wilcoxon rank-sum (Mann–Whitney U-test) test (for continuous variables) or Fisher’s exact test (for categorical variables). Using univariate and multiple logistic regression models, we analyzed the predictive role of platelets (thousands/mm^3^), ferritin (μg/L), transferrin saturation (TSAT) (%) in this high-risk population. Furthermore, we considered the three variables jointly, and we calculated the respective areas under the curve (AUC) of the receiver operating characteristic (ROC) curves. Analyses were performed with Stata 16 (StataCorp. 2019. Stata: Release 16. Statistical Software; StataCorp LP, College Station, TX, USA).

### Ethical aspects

The study was approved by the ethical review committee of the coordinating center “Comitato Etico Milano Area 2” (Protocol number 714/10) and by all participating Centers, and was carried out in compliance with the principles established in the Helsinki Declaration. Informed consent was obtained from all individual participants included in the study.

## Results

### The prevalence of GD1 in high risk predominantly Caucasian population is above 3%

**Figure 1:**
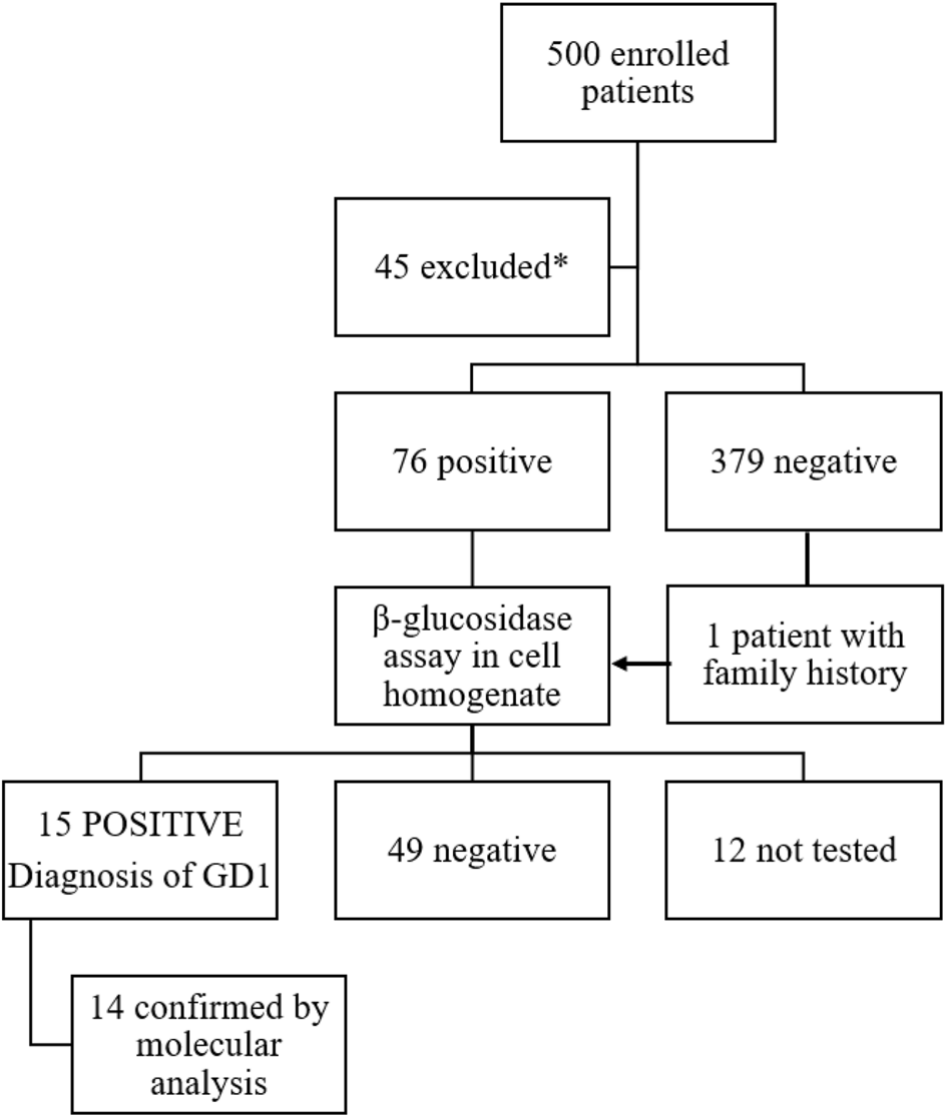
Results of DBS, β‐glucosidase assay in cell homogenate, and molecular analysis of the enrolled patients. *DBS* dried blood spot, *GD1* Gaucher disease type 1. *They did not fulfill the inclusion and exclusion criteria.

Five hundred subjects have been enrolled in the study. Forty-five have been excluded because they did not fulfill the inclusion and exclusion criteria (Fig. 1). Demographic, clinical, and laboratory characteristics are presented in Table 1. Ninety-one percent (91%) of the subjects were Caucasian. The mean age at enrolment was 46.9 years, and 31.9% (145/455) were females. The majority had splenomegaly (89.7%), and approximately half (47.9%) thrombocytopenia associated with other signs/symptoms. Anemia was the most common adjunctive sign (23.1%) (Table 1).

**Table 1:** Demographic, clinical, and laboratory characteristics.

Variable	Non-GD1 patients (n = 440)	GD1 patients (n = 15)	*p* value
Sex, F	138 (31.3%)	7 (46.7%)	0.26
Ethnicity C/J/O	400/2/38	14/0/1	0.28
Age, mean ± SD	47.0** ± **17.5	43.5 ± 15.1	0.38
**Inclusion criteria, n (%)**			
Splenomegaly	390 (88.6%)	14 (93.3%)	1.00
Splenomegaly alone	205 (46.6%)	4 (26.7%)	0.19
Thrombocytopenia	208 (47.3%)	10 (66.7%)	0.11
Thrombocytopenia alone	35 (7.9%)	1 (6.7%)	1.00
Anemia	101 (23.0%)	4 (26.7%)	0.75
Bone pain	67 (15.2%)	6 (40.0%)	0.02
MGUS	26 (6.0%)	2 (13.3%)	0.23
Polyclonal gammopathy U30	4 (0.9%)	0	1.00
Splenectomy	8 (1.8%)	0	1.00
**History of**			
Bone pain	76 (17.8%)	6 (46.2%)	0.02
Fractures	2 (0.5%)	0	1.00
Bleeding	22 (5.2%)	1 (7.1%)	0.53
Gallstones	52 (12.4%)	4 (26.7%)	0.11
Growth retardation	5 (1.2%)	1 (7.1%)	0.38
**Laboratory parameters**			
Hb, mean ± SD (g/dL)	13.3 ± 2.4	12.9 ± 2.1	0.28
WBC, mean ± SD (× 10^3^/mm^3^)	5.6 ± 2.3	4.5 ± 1.6	0.048
RBC, mean ± SD (× 10^6^/mm^3^)	4.8 ± 0.8	4.6 ± 0.6	0.20
PLT, median/range (× 10^3^/mm^3^)	131/8–767	84/44–148	**0.0006**
Iron, mean ± SD (μg/dL)	87.3 ± 44.7	76.5 ± 30.0	0.52
Ferritin, median/range (μg/L)	139/6–2858	551/55–1547	**0.0002**
Transferrin, mean ± SD (mg/dL)	234 ± 50	269 ± 51	0.03
TSAT, median/range (%)	25.7/5.9–87.2	20.8/7.2–26.7	0.03
ALT, median/range (UI/L)	29.8/1–496	25.7/1–57	0.98
AST, median/range (UI/L)	28.4/1–685	29.3/17–51	0.19
ALP, median/range (UI/L)	78.5/4–627	74.5/39–100	0.44
Cholesterol, mean ± SD (mg/dL)	157.7 ± 52.2	150.2 ± 31.8	0.84
**Serum protein electrophoresis**			
MGUS	17 (3.9%)	2 (13.3%)	0.12
**Bipolar spleen diameter, mean ± SD (cm)**	15.5 ± 2.4	17.1 ± 4.2	0.33

GD1: Gaucher disease type 1; C: Caucasian; J: Jewish; O: other; MGUS: monoclonal gammopathy of unknown significance; U30: under 30 years of age; Hb: hemoglobin: WBC: white blood count: RBC: red blood cells; PLT platelets; TSAT: transferrin saturation; ALT: alanine aminotransferase; AST: aspartate aminotransferase; ALP: alkaline phosphatase.

DBS showed normal values in 379 subjects, while 76 (16.7%) had a reduced β-glucosidase activity. These 76 patients and a patient with a family history of GD1 presenting with a β-glucosidase activity slightly above the lower normal range were recalled to test the conventional enzymatic activity. Among the 65 patients tested with β-glucosidase activity on nucleated cell homogenates (12 did not answer), 15 were diagnosed with Gaucher disease type 1, with a prevalence of 3.3% (15/455, 95% CI 1.9–5.4%). In 14, the molecular analysis of the *GBA* gene identified the mutations. In one patient, no mutations of *GBA* gene nor *PSAP*, encoding for saposin C, were identified. Among GD1 patients, 7/15 (46.7%) were female, 14 of Caucasian origin, and the mean age at diagnosis was 43.5 years. They showed a lower platelet count compared to non-GD1 patients (84.000/mm^3^ vs 131.000/mm^3^, *p* = 0.0006), a higher serum ferritin level (551 ng/dL vs 139 ng/dL, *p* = 0.0002) which was associated to a lower transferrin saturation (20.8% vs 25.7%, *p* = 0.03).

### Platelet count, ferritin, and transferrin saturation predict the probability of having GD1 in this high-risk population

**Figure 2:**
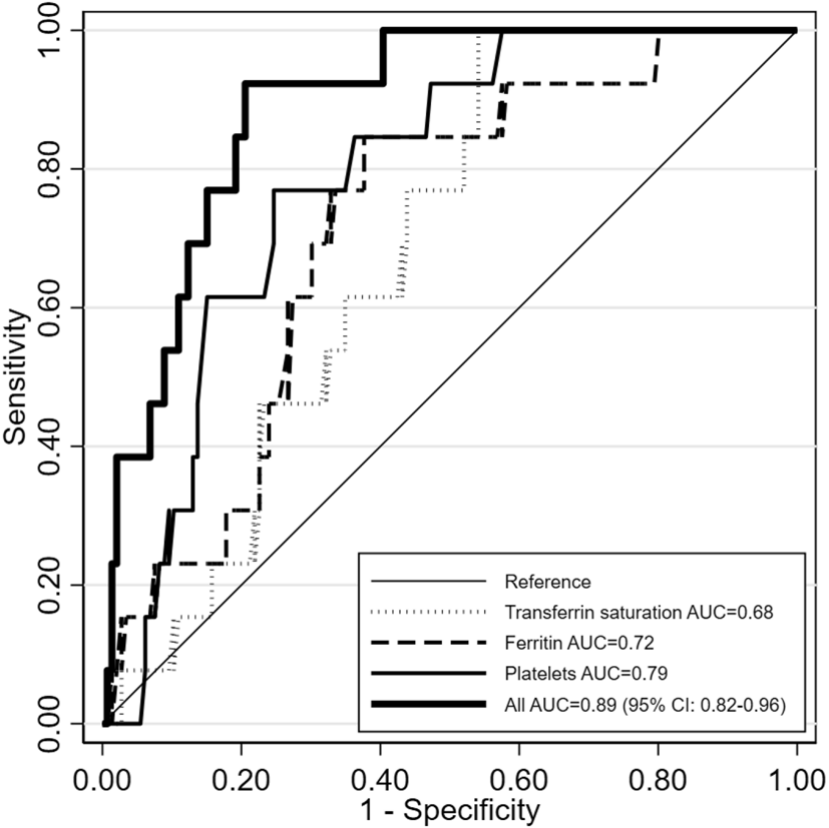
Receiver operating characteristic (ROC) curves of GD1 for platelets (thousands/mm^3^), ferritin (μg/L), transferrin saturation (%), and for the three variables jointly analyzed in a multiple logistic regression model. *AUC* area under the curve, *CI* confidence interval.

Considering 159 subjects (13 with GD1) with complete data on ferritin, platelets, and TSAT, the best discrimination between GD1 and non-GD1 subjects was provided by platelets (AUC = 0.79), while ferritin and transferrin saturation showed lower AUCs (Fig. 2).

The joint analysis of these three variables together in a multiple logistic regression model yielded an AUC of 0.89 (95% CI: 0.82–0.96). The corresponding equation is:

$${\text{odds}}\left( {{\text{GD1}}} \right) = {\text{exp}}\left[ {0.{311} + \left( {0.00{2} \times {\text{F}}} \right) + \left( { - 0.0{18} \times {\text{P}}} \right) + \left( { - 0.0{69} \times {\text{TS}}} \right)} \right]$$

where F = ferritin (μg/L), P = platelets (thousands/mm^3^), and TS = transferrin saturation (%).

Hence the predicted probability of GD1 in patients presenting with splenomegaly and/or thrombocytopenia plus the abovementioned ancillary signs can be calculated as: 100 × [odds(GD1)/[1 + odds(GD1)].]

When the probability predicted by the equation was < 5%, we observed only one subject with GD1 out of 93 (1.1%). When the predicted probability was 5 to < 10%, the observed frequency was 3.9% (1/26). With predicted probabilities ≥ 10%, the observed GD1 prevalence was substantially higher (11/30 = 27.5%).

The addition of a history of bone pain to this model did not lead to an AUC increase, and thus, this variable was omitted.

## Discussion

This study shows that in a predominantly Caucasian high-risk population presenting to the hematologist with splenomegaly and/or thrombocytopenia associated with other hematological signs, including anemia and MGUS, 3.3% of patients have Gaucher disease. These data confirm our previously published preliminary data^7^. Similar studies have been reproduced in different regions worldwide, with different prevalence, ranging from no cases detected in a Canadian study on 221 subjects^10^ to 7.0% in an adult cohort in China^11^. A similar approach is under evaluation in the Italian pediatric population^12^, and promising preliminary results have been presented in a Chinese study^13^. Altogether, these data support the use of the previously published algorithm by Mistry et al.^5^ associated with a simple first-level diagnostic test to screen high-risk populations. Diagnostic confirmation with β-glucosidase activity on nucleated cell homogenates is necessary to confirm the diagnosis. Of note, given that false negatives in the DBS test may arise due to methodological differences in blood spot drying, transport and storage^14–16^ testing with the gold standard diagnostic exam is warranted when there is any clinical suspicion of Gaucher disease, even in the presence of normal DBS values.

Since lysosomal storage disorders, including GD1, are underdiagnosed, other approaches have been proposed to increase the diagnostic rate. Namely, the new-born screening has been experimented in several regions with different results according to the ethnicity of the tested population^6,17–19^. However, the new-born screening raises unique issues that are primarily related to the inevitable detection of a disease with late-onset phenotypes.

Among the enrolled subjects, the only clearly different parameters between GD1 and non-GD1 patients were platelet count and serum ferritin. Hyperferritinemia with normal transferrin saturation is a common finding in naïve patients with GD1^20^, with prevalence ranging between 63 and 81%^21,22^. Recently, together with other Italian referral groups for iron disorders, we have proposed a new diagnostic flow-chart^23^, which enhances hyperferritinemia role when associated with splenomegaly and thrombocytopenia.

Here we propose an equation that predicts the probability of having GD1 according to platelet count and ferritin and TSAT levels and thus may support hematologists when evaluating a subject with splenomegaly and/or thrombocytopenia.

## Conclusion

High-risk population testing is effective in identifying Gaucher disease patients who present to the hematologist with splenomegaly and/or thrombocytopenia. The evaluation of the probability of having GD1 according to an equation and the use of DBS as a first-level test are potentially useful tools that can facilitate the diagnostic process.
